# Monitoring Apoptosis and Myeloid Differentiation of Acridine Orange‐Mediated Sonodynamic Therapy‐Induced Human Promyelocytic Leukemia HL60 Cells

**DOI:** 10.1002/jum.16575

**Published:** 2024-09-10

**Authors:** Metin Caliskan, Rahsan Ilikci‐Sagkan, Gulsen Bayrak, Sercin Ozlem‐Caliskan

**Affiliations:** ^1^ Department of Medical Biology, Faculty of Medicine Usak University Usak Turkey; ^2^ Department of Histology and Embryology, Faculty of Medicine Usak University Usak Turkey; ^3^ Department of Biophysics, Faculty of Medicine Usak University Usak Turkey

**Keywords:** acridine orange, differentiation therapy, HL60 cells, sonodynamic therapy

## Abstract

**Objectives:**

In the treatment of acute myeloid leukemia (AML), conventional therapies can lead to severe side effects and drug resistance. There is a need for alternative treatments that do not cause treatment resistance and have minimal or no side effects. Sonodynamic therapy (SDT), due to its noninvasive, multiple repeatability, localized treatment feature and do not cause treatment resistance, emerges as an alternative treatment option. However, it has not received sufficient attention in the treatment of AML especially acute promyelocytic leukemia (APL). The aim of the study was to investigate the potential differentiation and antileukemic effects of acridine orange (AO)‐mediated SDT on HL60 cells.

**Methods:**

Cell viability was determined by the 3‐(4,5‐Dimethylthiazol‐2‐yl)‐2,5‐Diphenyltetrazolium Bromide (MTT) method in the control, ultrasound, AO concentrations, and ultrasound‐exposed AO concentrations groups. Transmission electron microscopy (TEM) was used to determine morphology, and flow cytometry was used to determine apoptosis, DNA cycle, cell volume, mitochondria membrane potential (Δψm), reactive oxygen species (ROS) production, and differentiation markers (CD11b and CD15) expressions. Additionally, toluidine blue staining for semithin sections was used to determine differentiation.

**Results:**

The cytotoxicity of AO‐mediated SDT on HL60 cells was significantly higher than other groups, and TEM images showed that it caused various morphological changes typical for apoptosis. Flow cytometry results showed the presence of early apoptosis, subG1 arrest, loss of Δψm, increase of intracellular ROS production, decreased cell volume, and increased expression of CD11b (1.3‐fold) antigen and CD15 (1.2‐fold) antigen.

**Conclusion:**

Data showed that AO‐mediated SDT significantly induced apoptosis in HL60 cells. Increased expression of CD11b and CD15 antigens and morphological findings demonstrated that AO‐mediated SDT contributes to granulocytic differentiation in HL60 cells. AO‐mediated SDT has potential as an alternative treatment of APL.

**Abbreviations**
AML: acute myeloid leukemia
APL: acute promyelocytic leukemia
ATO: arsenic trioxide
FSC: forward scatter
PDT: photodynamic therapy
PI: propidium iodide
ROS: reactive oxygen species
SDT: sonodynamic therapy
TEM: transmission electron microscopy

Sonodynamic therapy (SDT) is a nonthermal treatment approach that employs low‐energy ultrasound to activate acoustically sensitive substances called sonosensitizer, inducing the death of tumor cells while minimizing harm to normal tissue.^1^, ^2^ Metabolic dysfunction in tumor cells causes relative accumulation of sonosensitizer, resulting in higher cytotoxic effect in tumor cells.^1^ Furthermore, neither ultrasound nor sonosensitizer alone is harmful. Sonosensitizer has a significant cytotoxic effect in the area where ultrasound is applied only with the presence of sufficient molecular oxygen.^3^ Additionally, this noninvasive treatment can be repeated many times and does not cause any treatment resistance.^4^, ^5^, ^6^ Having all these advantages over classical cancer treatments, SDT has become a candidate for adjunctive and/or alternative treatment in recent years. SDT is a noninvasive, alternative cancer treatment method developed based on the principles of photodynamic therapy (PDT). While PDT uses light to activate the sensitizer, SDT uses ultrasound instead.^7^

Sonodynamic research on SDT commenced in 1989, with Umemura et al reporting that a combination of ultrasound and hematoporphyrin, a photosensitizer, exhibited cytotoxic effects on mouse sarcoma cells.^8^ Subsequent studies have revealed that porphyrin‐based photosensitizers, akin to those used in PDT, display similar efficacy when combined with ultrasound.^9^, ^10^, ^11^, ^12^, ^13^ SDT surpasses PDT in terms of tissue penetration, capable of reaching depths of several centimeters, contingent upon the chosen ultrasound frequency, thus offering access to deeper‐situated tumors.^14^

SDT employs multiple mechanisms to destroy targeted cells, encompassing the oxygen radical theory, cavitation effect theory, induction of apoptosis, promotion of antitumor immunity, inhibition of angiogenesis, and induction of hyperthermia.^15^, ^16^, ^17^ These specific effects vary depending on the intensity and frequency of the ultrasound. High‐intensity sonication leads to heat generation, while low‐frequency operation increases the likelihood of cavitation, which refers to the formation of microbubbles due to pressure fluctuations during ultrasound propagation in tissue fluid.^11^ Exposure of biological tissues to ultrasound can result in both functional and structural changes in cells.^18^ The duty cycle also plays a critical role in heating. The value of the duty cycle indicates what percentage of the time the ultrasonic signal is produced. As the pulse length decreases, a reduction known as the duty factor occurs. Consequently, the thermal effect is suppressed.^19^

Upon exposure to ultrasound at a specific frequency and intensity, sonosensitizers undergo an energy transition, moving from the ground state to the excited state. Subsequently, as the excited electron returns to the ground state, the generation of reactive oxygen species (ROS) leads to cell death through lipid peroxidation.^20^ Following SDT, apoptosis is induced through molecular mechanisms.^17^, ^18^, ^20^, ^21^ The primary mechanisms believed to underpin SDT's ability to eliminate target cells involve the initiation of lipid oxidation through ROS, target cell destruction through the cavitation effect, and the induction of apoptosis.

Acridine orange (AO) is a water‐soluble photodynamic fluorochrome that can be excited by light.^22^, ^23^, ^24^ AO can diffuse into cell cytoplasm within a few seconds due to its low molecular weight. Being a basic dye, AO readily binds to other acidic cellular structures, including DNA, RNA, and lysosomes.^25^ When excited by photon energy from visible light, AO generates reactive singlet oxygen molecules.^24^, ^25^, ^26^ Numerous experimental studies have demonstrated AO's photosensitizing properties, making it a valuable component in PDT for cancer treatment.^27^, ^28^, ^29^, ^30^, ^31^, ^32^ However, the effect of AO‐mediated SDT on HL60 cells has not yet been defined. Moreover, not only cytotoxicity but also differentiation is important for treatment. For this reason, we chose to use the promyelocytic leukemia model cells, the HL60 cell line, in our study.

Although differentiation therapy is the leading option in the treatment of acute promyelocytic leukemia (APL) today and offers highly successful treatment, relapses resulting from chemoresistance increase the need for alternative or combined treatments.^33^ This study aims to examine the effects of AO‐mediated SDT on HL60 cells from multiple perspectives, including cytotoxicity (MTT proliferation test), morphological changes (Giemsa staining and transmission electron microscopy [TEM]), apoptosis induction (Annexin V/PI with flow cytometry, mitochondrial membrane potential analysis, cell cycle analysis, and ROS analysis), as well as differentiation (CD11B and CD15) in HL60 cells.

## Materials and Methods

### Cell Culture

HL60 acute promyelocytic leukemia cells were cultivated in RPMI 1640 medium with L‐glutamine, supplemented with 10% fetal bovine serum and 1% penicillin/streptomycin. The cultures were maintained in a humidified environment with 5% CO₂ at 37°C. All cell maintenance and culture preparation procedures were conducted under sterile conditions within a laminar flow cabinet.

### Chemical

In this study, acridine orange zinc chloride double salt (AO) (AppliChem) with a molecular weight of 438.12 g/mol was used as a sonosensitizer. An AO stock solution was prepared using phosphate buffered saline (PBS), and various AO concentrations of 0.0625, 0.125, 0.25, 0.5, 1, 2, and 4 μM were created using RPMI medium. The maximum absorption of the stock solution was detected at 464 nm.

### Determining the In Vitro Efficacy of SDT

HL60 cells (5 × 10^5^ cells/mL) were incubated with AO concentration solutions for 40 minutes at 37°C in the dark. After incubation, HL60 cells were centrifuged at 200× g for 5 minutes. The resulting pellet was washed three times with PBS to eliminate any remaining AO, and fresh medium was added. Ultrasound was administered using the BTL 4710 Sono dual‐frequency ultrasound therapy device (BTL, CZ). Cells were exposed to the near field of a horizontal continuous wave ultrasound beam at an acoustic power of 1.4 W and an output intensity of 2 W/cm^2^ for 3 minutes in 1.5 mL Eppendorf tubes immersed in degassed water at a frequency of 1 MHz.^34^ Subsequently, the cells were incubated at 37°C for 24, 48, and 72 hours after the ultrasound treatment.

### Cell Viability Analysis

MTT is a colorimetric assay for indirectly determining cell viability by measuring cellular metabolic activity. The viability of HL60 cells was assessed using the Cell Proliferation Kit I (MTT) (Roche Diagnostic, Germany) according to the manufacturer's instructions. MTT labeling reagent was added at post‐treatment 24, 48, and 72 hours and then incubated at 37°C for 4 hours. Following incubation, a solubilization buffer was introduced, and the mixture was kept in an oven at 37°C overnight. Cell viability was measured using a Multiskan Sky Microplate Spectrophotometer, a microplate reader from Thermo Fisher Scientific, at wavelengths ranging from 550 to 600 nm according to the manufacturer's instructions.

### Transmission Electron Microscope Analysis

For electron microscopic examinations, HL60 cells were centrifuged at 1500 rpm for 5 minutes. To the pellet, 2.5% glutaraldehyde was added and allowed to stand for 10 minutes. Subsequently, the cells were centrifuged at the 21,000× g for 5 minutes, followed by the addition of another 2.5% glutaraldehyde and incubation for 20 minutes. After 20 minutes, the pellet was removed from the tube, cut into 1‐mm 3 pieces and washed with PBS. The pellet pieces were kept in PBS at +4°C until tissue processing was done. After standard tissue processing for electron microscopy, the cells were embedded in epoxy resin and blocked. Thin sections of 2000 nm were obtained from the blocks and stained with toluidine blue. Suitable areas were chosen for the thin sections. Ultra‐thin sections with a thickness of 70 nm were obtained using a ultramicrotome (Leica UCT125, Leica GmbH, Germany) from the block, and contrasted with uranyl acetate and lead citrate for 5 minutes. The sections were examined and photographed using a transmission electron microscope (TEM, Jeol JEM 1400).

### Apoptosis Detection Using Annexin V/PI Staining

In apoptotic cells, the membrane phospholipid phosphatidylserine migrates from the inner surface of the plasma membrane to the outer surface. Annexin V dye specifically binds to phosphatidylserine, allowing for the early detection of cells in the initial stages of apoptosis. Propidium iodide (PI) is applied to stain late apoptotic, and necrotic cells. The impact of various treatments, including control, US, AO only, and AO‐mediated SDT, on apoptosis was assessed using the Annexin V‐FITC/PI test. Cells were stained with Annexin V‐FITCH (1 μL) and PI (5 μL) for 15 minutes in the dark. Apoptosis induced by AO‐mediated SDT in HL60 cells was analyzed using flow cytometry in a population of 10,000 cells, and the percentage of viable, necrotic, early and late apoptotic cells was calculated.

### SubG1 Analysis

The subG1 peak analysis is a technique used to identify cells that have undergone DNA loss in the late stages of apoptosis. Cellular DNA content was assessed using the BD Cycle‐test™ Plus DNA Reagent Kit (BD Biosciences, USA). Samples were incubated in a dark room with Solution A, containing trypsin buffer and trypsin inhibitor, Solution B, containing RNase buffer, and Solution C, containing PI dye, for 10 minutes each. The DNA content of the cells was then analyzed using BD Acquire C6 flow cytometry. The percentage of apoptotic cells was determined through subG1 peak analysis in HL60 cells.

### Determination of Cell Volume

Measuring the average cell volume is a means to assess cytotoxic effects on the cell membrane. Cell volume reduction is another method of determining the process of apoptosis. Changes in cell volume are detected through the assessment of forward scatter (FSC) values using flow cytometry. Cell volume for all groups was determined by analyzing the FSC‐H calculation in histogram‐type charts.

### Mitochondrial Membrane Potential (Δψm)

One of the techniques to assess any potential mitochondrial damage is the measurement of ∆Ψm conducted via flow cytometry using JC‐1 dye (BD Biosciences, USA). This technique allows the detection of mitochondrial depolarization associated with cell death. Mitochondrial depolarization is characterized by a decrease in red fluorescence intensity and a corresponding increase in green fluorescence intensity. ∆Ψm values of HL60 cells were determined after various treatments, including control, US‐control, AO only, and AO‐mediated SDT. After HL60 cells were centrifuged at 300× g for 5 minutes, JC‐1 working solution was added and incubated at 37°C for 15 minutes. After 15 minutes, cells were washed twice with JC‐1 buffer. ∆Ψm values were measured using the BD Acquire C6 Plus flow cytometry.

### Measurement of ROS

Total ROS encompass chemically reactive oxygen‐containing molecules produced as natural by products of oxygen metabolism. Examples of ROS include superoxide ions and peroxides. To investigate whether ROS release occurred, the ROS measurement test was applied to the control, and AO‐mediated SDT groups. The ROS measurement was conducted following the experimental protocol of the ROS assay kit (Invitrogen Thermo Fisher Scientific, UK). H_2_DCFDA probe was added to the samples and incubated for 60 minutes. Subsequently, ROS activity within the cells was quantified using BD Acquire C6 Plus flow cytometry.

### Analysis of Myeloid Differentiation

#### Toluidine Blue Staining for Semithin Sections

Thin sections stained with toluidine blue were used to examine changes in nuclear morphology in nonapoptotic cells. For this, the nuclei of 100 differentiated cells were classified as ovoid, indented, lobulated, and multilobed at 1000× magnification.^35^ All examinations were observed by using a light microscope (Nikon ECLIPSE E200) and photographed.

#### CD11b and CD15 Expression Analyses of Granulocytic Differentiation

Control, US, AO, and AO‐mediated SDT‐treated HL60 cells washed with PBS. PE‐conjugated antihuman CD11b and FITC‐conjugated antihuman CD15 antibodies (BD Biosciences, Ireland) was subsequently added to HL60 cells and incubated for 30 minutes. Following incubation, cells were washed with PBS and analyzed using a Becton Dickinson Accuri CPlus flow cytometer (BD Biosciences, USA). Untreated cells were also used to check for nonspecific binding. Results were recorded as the mean fluorescence intensity value for flow cytometric analysis.

### Statistical Analysis

The experiments were conducted in triplicate, with each experiment having duplicate measurements. The results were automatically determined as mean ± standard deviation (SD) and the graph was drawn for data presentation and analysis by SPSS version 25 software. One‐way analysis of variance, followed by the Tukey post hoc test, as well as independent sample *t* tests were used for the evaluation of *P*‐value. Significance was considered at *P* < .05 for pairwise comparisons, *P* < .008 for 4‐way comparisons, and *P* < .005 for 5‐way comparisons.

## Results

### Cytotoxic Activity of AO‐Mediated SDT

**Figure 1:**
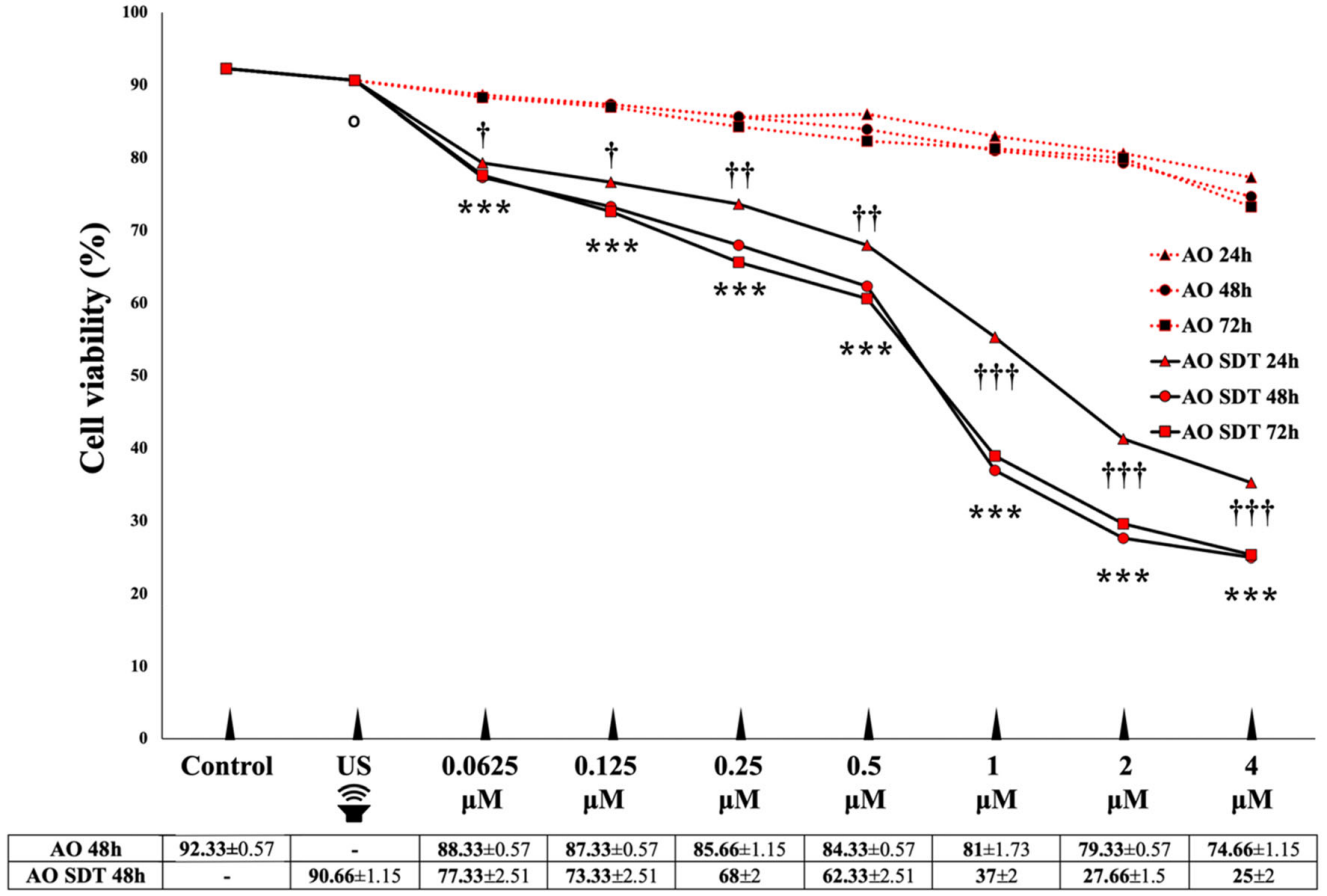
Evaluation of cytotoxicity after treatment with AO and AO‐mediated SDT. Graphic representation of viability of HL60 cells at 24, 48, and 72 hours demonstrating the enhanced effect of combining AO and US. In order to show the decrease in cell viability caused by experimental conditions such as ultrasound, centrifugation and PBS washing, the viability of cells that were not exposed to experimental conditions and not removed from the incubator throughout the experiment was accepted as 100%. Statistical significances were obtained by comparing all concentrations of the AO SDT group with control, US, and all concentrations of the AO groups. ***P < .001, ^ο^ *P* > .05 (comparisons between groups), ^†^ *P* < .05, ^††^ *P* < .01, ^†††^ *P* < .001 (comparisons between AO SDT group 24, 48, and 72 hours incubation periods). All *P*‐values are presented in Supplement S1.

The viability percentages of the control, US, AO, and AO‐mediated SDT groups at 24, 48, and 72 hours are illustrated in Figure 1. The results showed that exposure to AO‐mediated SDT at 48 hours led to a substantial reduction in the viability of HL60 cells. Specifically, cell viability in the AO‐mediated SDT group, across all concentrations, was markedly lower compared to the AO, US, and control groups at equivalent concentrations (*P* < .001). Cell viability results reveal that AO‐mediated SDT significantly heightened cytotoxicity in HL60 cells. When compared with the control group, no statistical significance was found in the US group (*P* > .05), whereas all AO SDT groups were found to be statistically significant compared to the control and US groups. There was no significant difference detected among the 24‐, 48‐, and 72‐hour incubation periods in the AO group at the same concentration (*P* > .05). While there was no significant difference between the 48‐ and 72‐hour incubation periods at the same concentrations of the AO SDT group (*P* > .05), it was determined that the 24‐hour incubation period at the same concentrations showed statistically significant cell viability compared to both the 48‐ and 72‐hour incubation periods (0.0625 and 0.125 μL, *P* < .01; other AO concentrations, *P* < .001). Among these incubation period, it was observed that the AO‐mediated SDT effect did not fully occur at 24 hours, while the full effect occurred very close to each other at 48 and 72 hours. It was decided that 48 hours which is shorter incubation period, would be preferred to be used in the further stages of the experiment. The IC_50_ value was established as 1 μM, and subsequent experiments were conducted with the IC_50_ value.

### Electron Microscopy Findings

**Figure 2:**
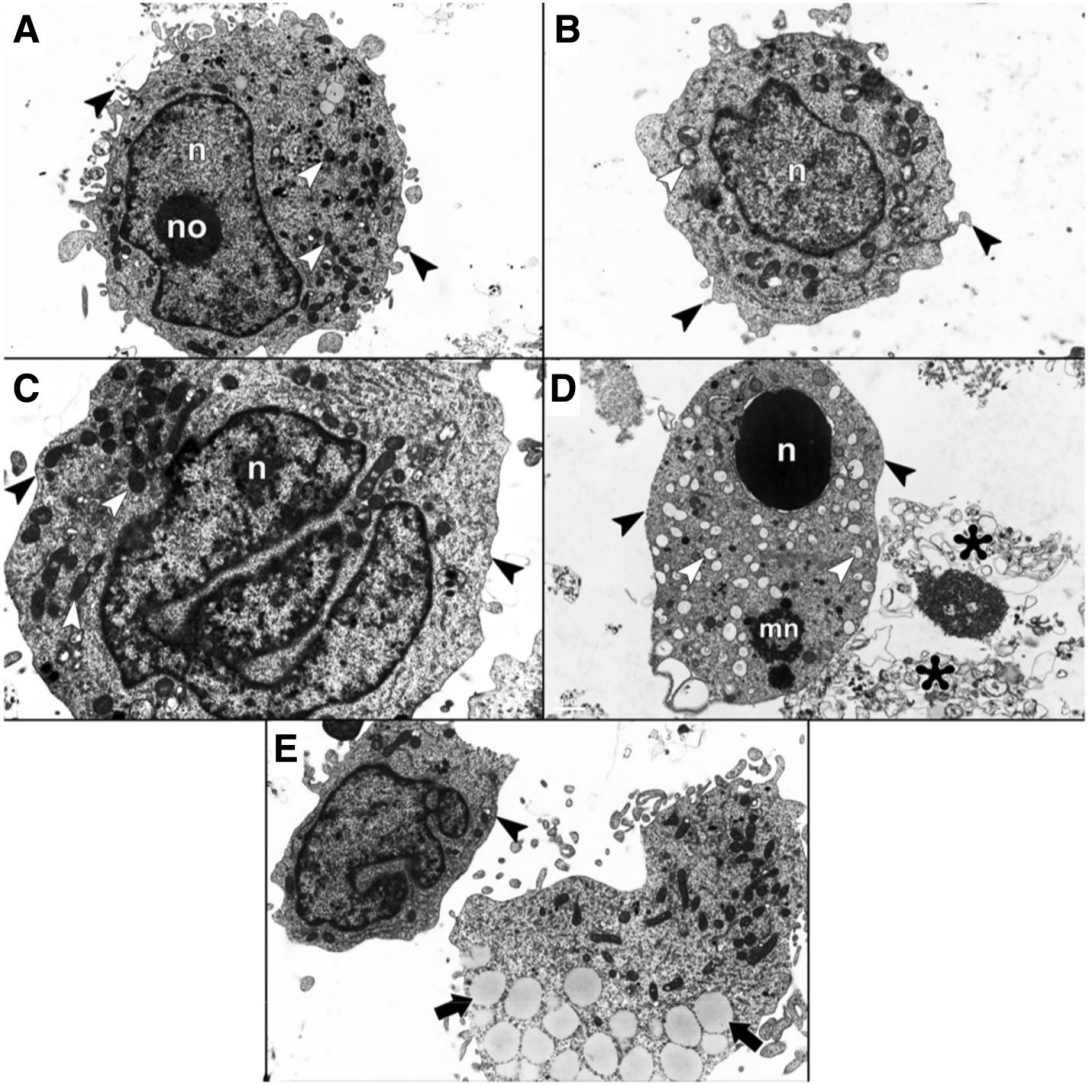
Electron microscopic photomicrograph of control (**A**), US (**B**), AO (**C**), and AO‐mediated SDT (**D** and **E**) groups. In the control group had normal ultrastructural nucleus (n), nucleolus (no), mitochondria (white arrowhead), and short microvilli (black arrowhead). Normal nucleus (n) and short microvilli (black arrowhead); loss of cristae in mitochondria (white arrowhead) in the US group. Lobulation in the nucleus (n), normal mitochondria (white arrowhead), and decreased microvilli (black arrowhead) in the AO group. In the AO‐mediated SDT group chromatin condensation and multilobulation in the nucleus (n) and micronuclei (mn), loss of cristae, and degenerative changes in the mitochondria (white arrowhead), loss of microvilli on the surface of the cells (black arrowhead), cell debris in the intercellular space (black asterisk), and increased lipid droplets (black arrow). **A**, **B**, and **D**: ×12,000; **C:** ×15,000; **E**, ×8000.

Examination of the control group with TEM revealed cell nuclei, mitochondria, and other organelles with normal ultrastructure. Chromatin was uniformly distributed within the nuclei, and the nucleolus was readily visible. The cytoplasm contained numerous mitochondria with parallel cristae. Furthermore, short microvilli were present on the cell's outer surface (Figure 2A). In the US group, cell nuclei appeared normal, with uniformly distributed chromatin. However, some cells displayed a loss of mitochondrial cristae. The cell surface maintained the presence of normal microvilli (Figure 2B). The AO group exhibited cells with nuclei, some of which displayed lobulation, in addition to cells with normal nuclei. The mitochondria and other organelles retained their typical morphology. There was a reduction in the number of short microvilli on the cell surface when compared to the control group cells (Figure 2C). In the AO‐mediated SDT group, distinct chromatin condensation and marginalization were evident in the nuclei. Furthermore, some cells showed nuclear lobulation and the presence of apoptotic micronuclei. Lytic cell remnants were observed in numerous areas. Many cells displayed intracellular vacuoles within the cytoplasm. Mitochondria exhibited a loss of cristae in many cells, along with vacuoles and degenerative changes. There was also a marked decrease in the number of short microvilli on the cell surface in many cells. Additionally, lipid droplets were significantly increased in this group in comparison to the other groups (Figure 2, D and E).

### Annexin V/PI Staining

**Figure 3:**
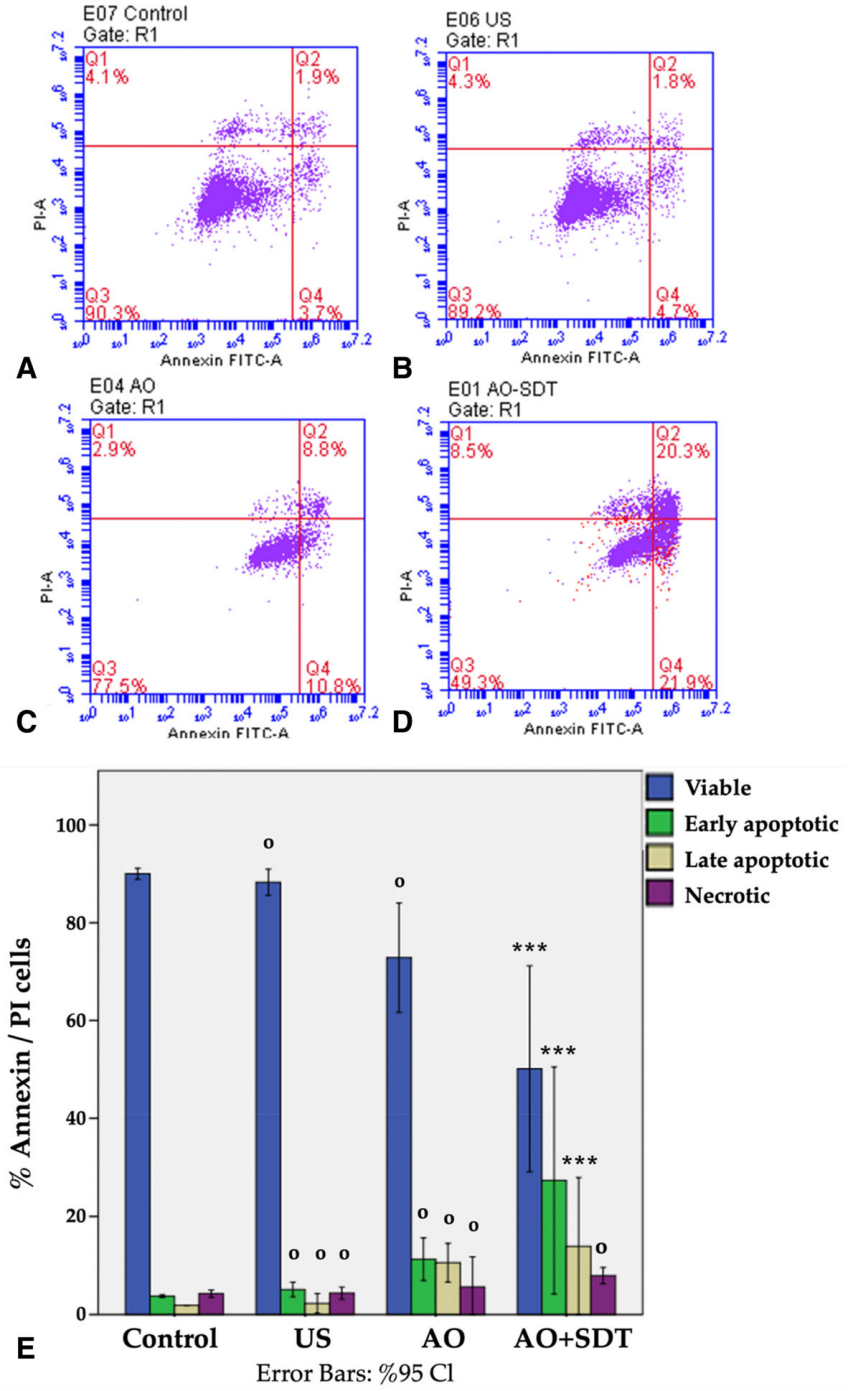
HL60 cells, untreated (**A**) and treated with US (**B**) and treated with AO (**C**) and treated with AO‐mediated SDT (**D**) were stained with Annexin V‐FITC and PI and analyzed by flow cytometry. The dot plots are representative of one of the 3 independent experiments. (**E**) Bar graphs representing mean % Annexin V/PI cells. ***P < .001. *** indicates statistically significance compared to control group. ^ο^ *P* > .05. ^ο^ indicates not statistically significance compared to control group.

To decipher the mechanism of cell death induced by AO‐mediated SDT, HL60 cells were assessed using Annexin V‐FITC/PI staining. The histogram was divided into 4 quadrants, with each quadrant representing a distinct cell population. The first quadrant, Q1, represents only dead cells stained with PI (Annexin V^−^/PI^+^). The second quadrant in the scatter plot, Q2, depicts cells that are both Annexin V and PI stained, indicating late apoptotic and necrotic cells (Annexin V^+^/PI^+^). The third quadrant, Q3, depicts live cells (Annexin V^−^/PI^−^), and the fourth quadrant, Q4, depicts early apoptotic cells stained only with Annexin V (Annexin V+/PI^−^). In the control group, approximately 90% of cells were detected in Q3 (Figure 3A). In the US group, a similar cell population was observed in Q3 when compared to the control group (88.3%, Figure 3B). In the AO and AO‐mediated SDT groups, 72.8% and 50.1% of cells were found in Q3, respectively (Figure 3, C and D). The percentage of cells treated with AO‐mediated SDT in the Q4 quadrant, representing early apoptotic cells, was approximately 24.3% (Figure 3D). Furthermore, AO‐mediated SDT of HL60 acute myeloid leukemia cells led to an increase in the percentage of late apoptotic cells (Q2) compared to the control and US groups (1.9%, 2.2%, 13.9%; Figure 3, A–C). The percentage of necrotic cells (Q1) in the control, US, and AO groups was 4.2%, 4.3%, and 5.6%, respectively, while it was 7.9% in the AO‐mediated SDT group. The percentage values obtained from Annexin V/PI staining for early apoptotic, late apoptotic, and live cells showed significant differences in favor of AO‐mediated SDT when compared with the control group (***P < .001, Figure 3E). Statistically, it was determined that early and late apoptotic cells also significantly changed in the AO‐mediated SDT group compared to the US group (***P < .001). However, no statistically significant change was detected in the group treated with AO alone. No difference or significance was found between the groups in terms of necrotic cells either (Supplement S1). Additionally, these results confirm that AO‐mediated SDT induces early apoptosis in HL60 cells.

### Cell Cycle Analysis in HL60 Cells After SDT

**Figure 4:**
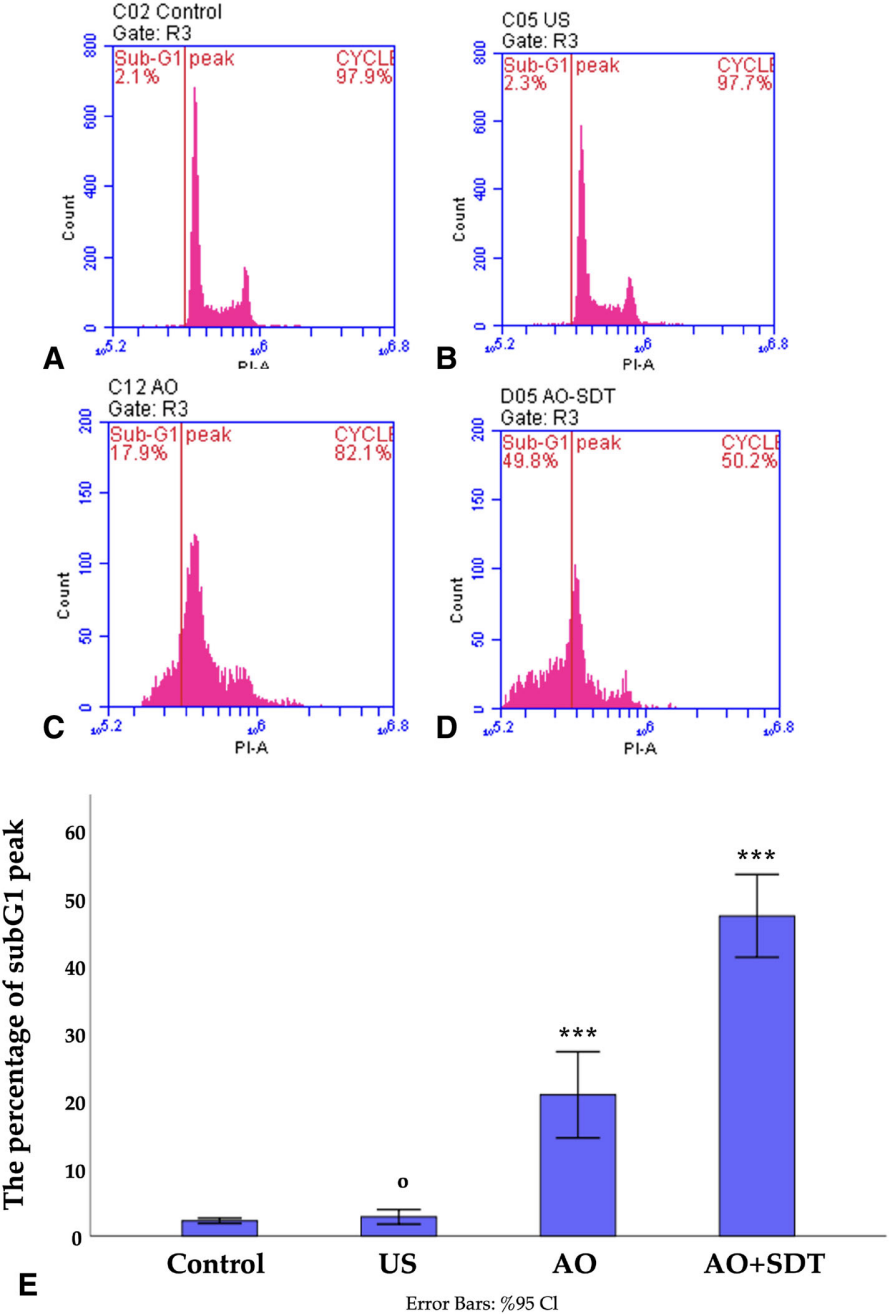
SubG1 apoptotic peak was determined by flow cytometry. Flow cytometry histograms. (**A**) Control, untreated HL60 cells; (**B**) control, treated with US; (**C**) HL60 cells treated with AO; (**D**) HL60 cells treated with US combined with AO; (**E**) graphical presentation of the percentage of the subG1 peak apoptotic cell. *** indicates statistically significance compared to control group; ^ο^ indicates not statistically significance compared to control group. ***P < .001, ^ο^ *P* > .05.

The control, US, AO, and AO‐mediated SDT groups were subjected to PI staining and analyzed using flow cytometry 48 hours after treatment. The amount of fluorescence intensity is directly related to the DNA content in cells, and DNA degradation in apoptotic cells results in a lower PI intensity compared to G1 phase cells (subG1 peak). After 48 hours of treatment, a significant increase indicating apoptosis in the subG1 peak (47%) was observed in the AO‐mediated SDT group with an IC_50_ dose. While only 2.2% of cells in the control group were detected in the subG1 peak region, cells in the US and AO groups were found to be 2.8% and 20.8% in the subG1 peak region, respectively (Figure 4, A–E). The results obtained from this analysis indicate that AO‐mediated SDT with the IC_50_ dose can halt the cell cycle of HL60 acute myeloid leukemia cells at the subG1 level. This suggests that AO‐mediated SDT can induce cell apoptosis.

### Reduction in Cell Volume Following AO‐Mediated SDT

**Figure 5:**
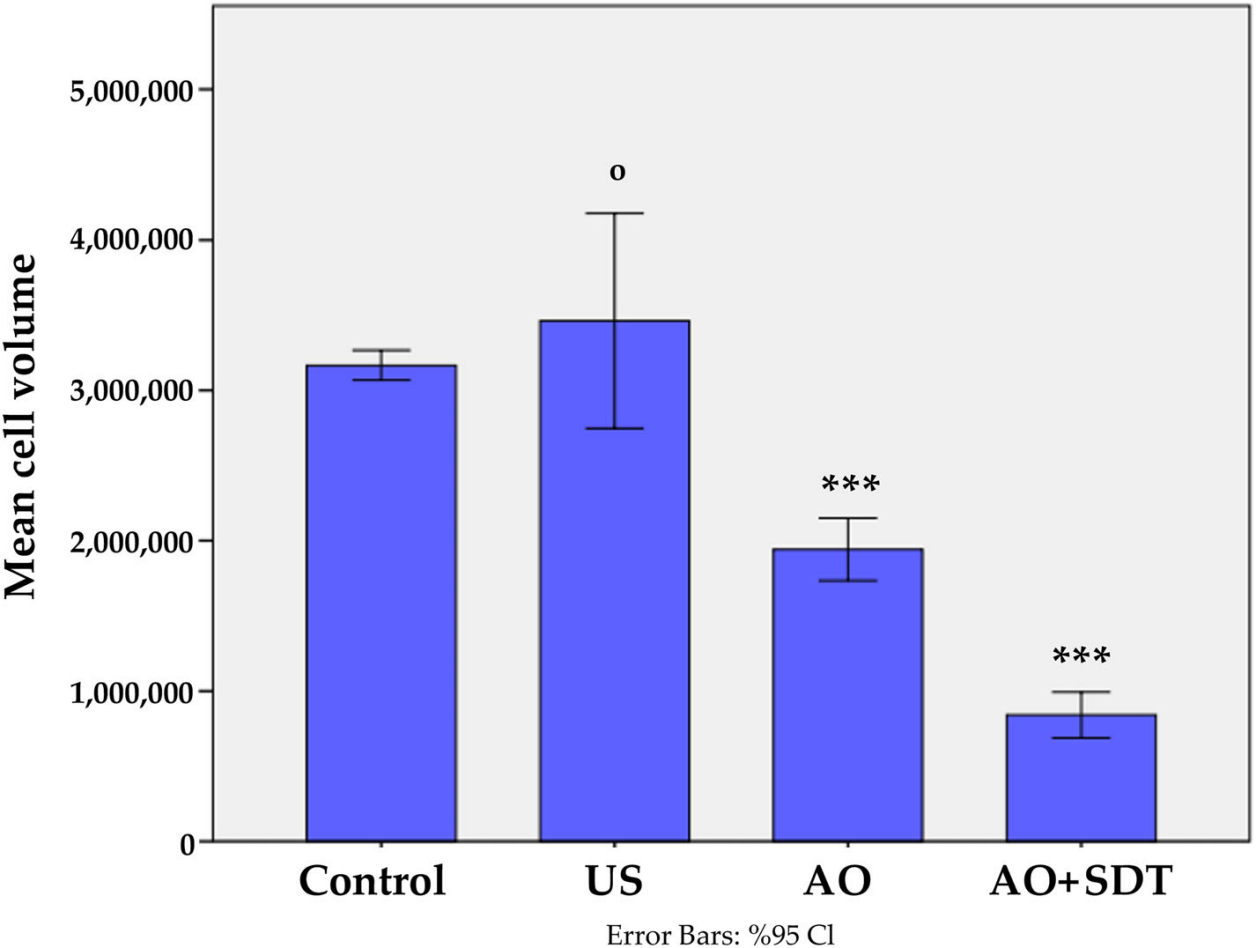
Cell volume analysis of HL60 treated with AO and AO‐mediated SDT. Bar graphs representing mean % cell volume analysis. *** indicates statistically significance compared to control group; ^ο^ indicates not statistically significance compared to control group. ***P < .001, ^ο^ *P* > .05.

A reduction in cell volume is another method to determine the apoptosis process that can often be quantified using flow cytometry. In Figure 5, it is observed that there is a 38.7% decrease in cell volume in cells treated with AO compared to the control group, whereas cells treated with AO‐mediated FDT show a 73.5% decrease in cell volume. The values obtained from cell volume measurements in the AO and AO‐mediated SDT groups exhibited a significant difference compared to the control group (***P < .001, Figure 5).

### Changes in Δψm

**Figure 6:**
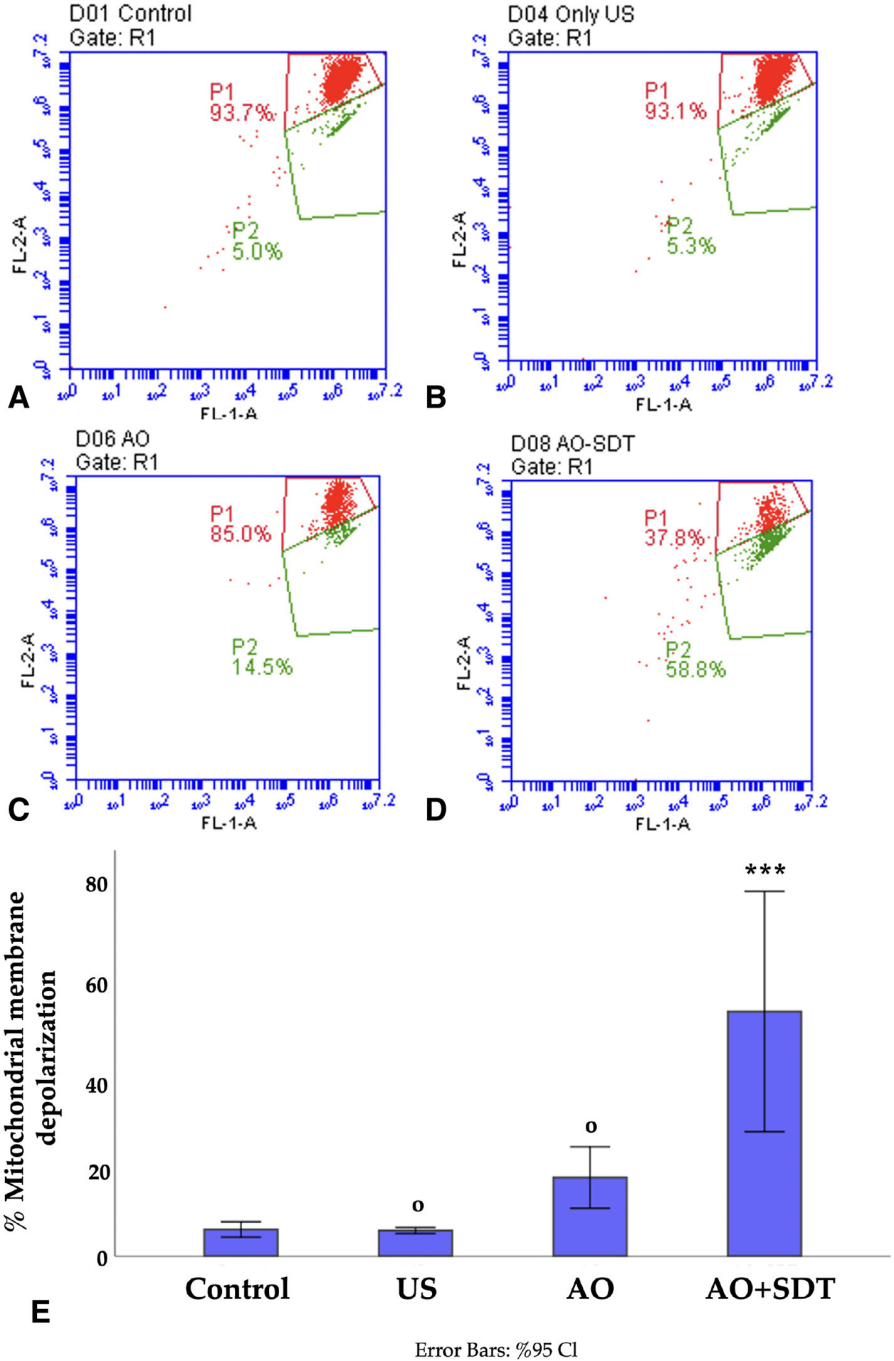
Changes in the ΔΨm of HL60 cells after 48 hours of incubation with AO, AO‐mediated SDT. (**A**) Control; (**B**) treatment with US; (**C**) treatment with AO; (**D**) treatment with AO‐mediated SDT. The dot plots are representative of 3 independent experiments. (**E**) Bar graphs representing mean % green fluorescent cells. Results are presented as means + SD; *n* = 3 ^ο^ *P* > .05, ***P < .001. ^ο^ indicates not statistically significance compared to control group, *** indicates statistically significance compared to control group.

JC‐1 fluorescence was assessed by determining the percentage of cells in 2 distinct populations, namely P1 and P2, as illustrated in Figure 6. In this context, P1 represents mitochondrial resting membrane potential, while P2 denotes depolarized mitochondrial membrane and cells with potential susceptible to apoptosis. Mitochondrial depolarization is characterized by a decrease in red fluorescence intensity and a corresponding increase in green fluorescence intensity. In the control, US, and AO groups (Figure 6, A–C), higher cell populations were observed with red fluorescent signals (93.7%, 93.1%, 85%), whereas in the AO‐mediated SDT group (52.7%), an approximately eightfold stronger green fluorescent signal was observed compared to the control and US groups (5.7%, 5.5%) (Figure 6D). The percentage of mitochondrial membrane depolarization in the control, US, AO, and AO‐mediated SDT‐treated cells was 5.7%, 5.5%, 16.9%, and 52.7%, respectively (Figure 6E). A statistically significant difference was found when the cell group treated with AO‐mediated SDT was compared with the control, US, and AO groups (*P* < .001). The mitochondrial depolarization in the US and AO groups did not show a significant increase compared to the control group. No statistically significant difference was found (*P* = 1.000, *P* = .125).These results confirm the induction of apoptotic cell death following AO‐mediated SDT in HL60 cells, supported by the observed alterations in mitochondrial membrane potential.

### Measurement of Intracellular ROS

**Figure 7:**
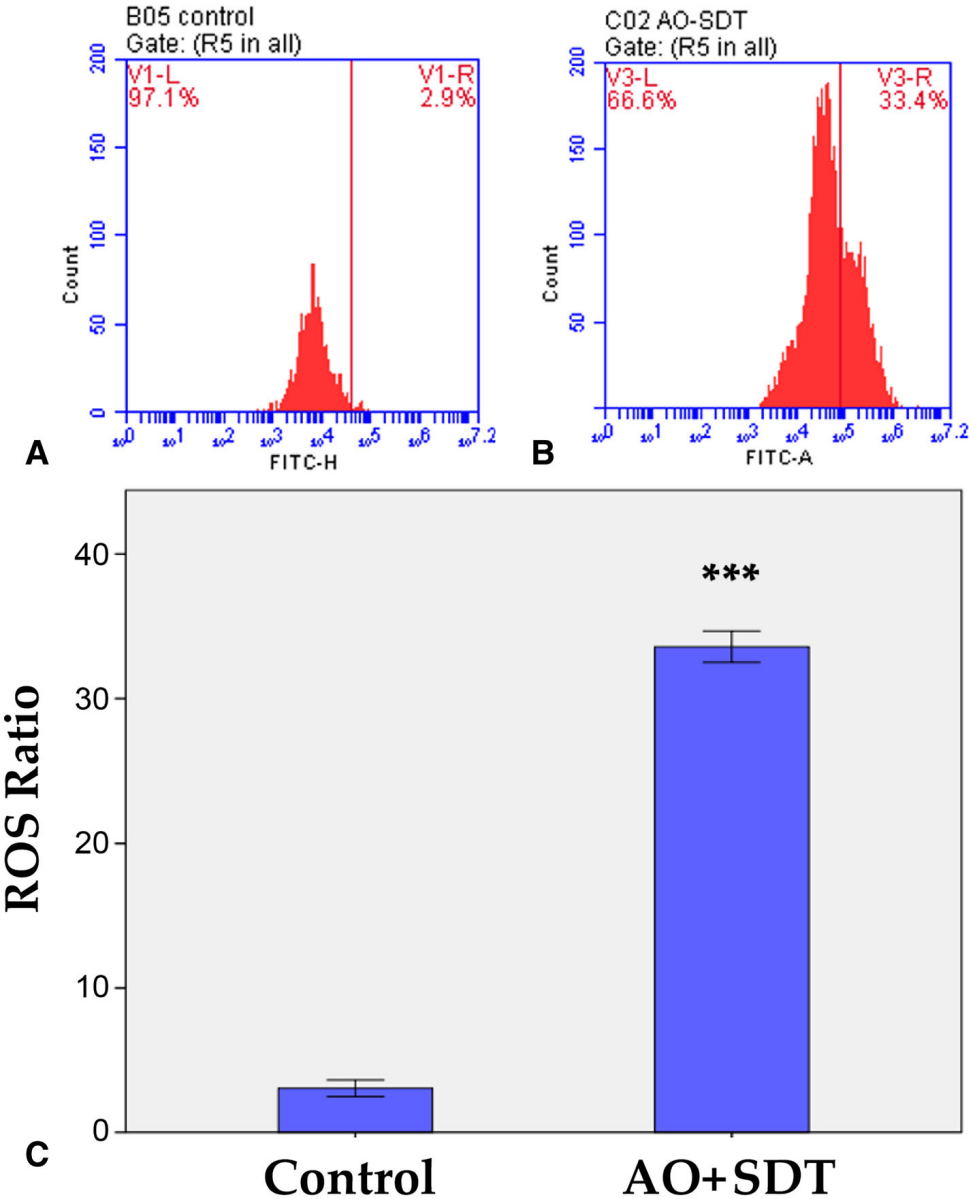
Effect of AO‐mediated SDT on excessive ROS generation in HL60 cells. ROS was measured by staining the cells with H_2_DCFDA cellular ROS detection assay kit according to the manufacturer's instructions. ROS generation was measured by flow cytometer. (**A**) Control, untreated HL60 cells; (**B**) HL60 cells treated with AO‐mediated SDT; (**C**) graphical presentation of the ratio of ROS generation in HL60 cells. Graphical results are given as mean + SD of three experiments, ***P < .001. *** indicates statistically significance compared to control group.

To determine whether AO‐mediated SDT cell apoptosis occurs through ROS production, intracellular ROS levels were determined using H_2_DCFDA probe. AO‐mediated SDT significantly increased ROS generation in HL60 cells compared to control. In the AO‐mediated SDT group, an approximately 11.2‐fold increase in ROS fluorescence was observed when compared to the control group (Figure 7, A and B). Consequently, Figure 7C demonstrates that AO significantly (*P* < .001) enhances ROS production in HL60 cells when excited with SDT. These results indicated that ROS production might be the possible cause of the apoptosis caused by the combination of AO and SDT in acute promyelocytic leukemia cells.

### Examination of Morphology With Toluidine Blue and Analysis of CD11b and CD15 Expression by Flow Cytometry

**Figure 8:**
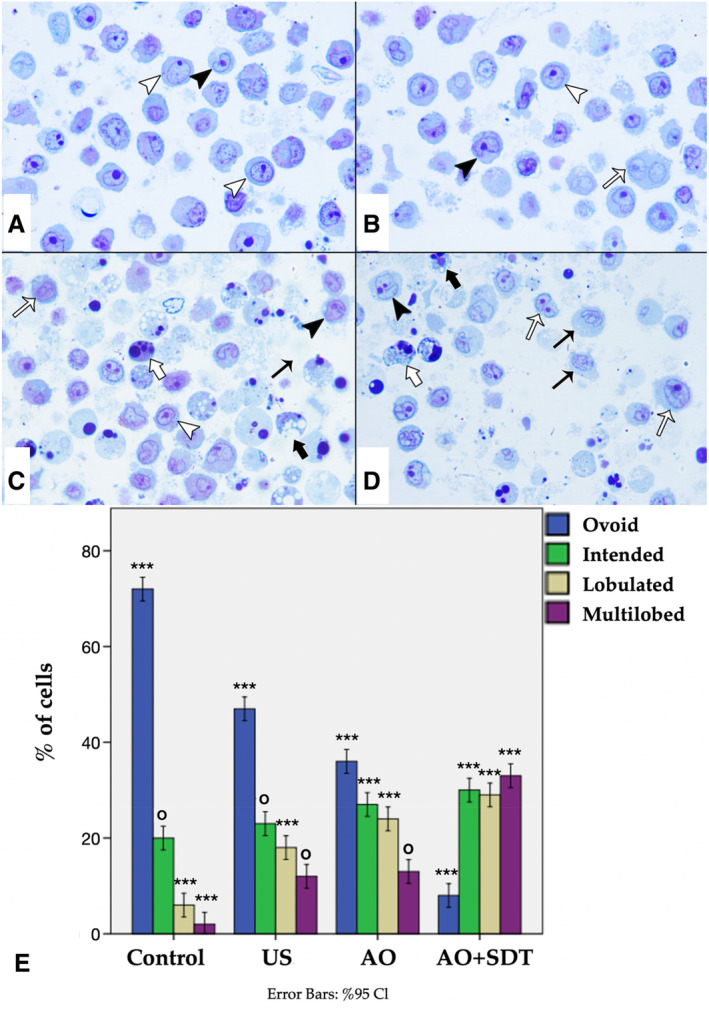
Toluidine blue staining. Control (**A**), US (**B**), AO (**C**), and AO‐mediated SDT (**D**). (**E**) Percent of cells in each category; the data are shown as the mean ± SD; n = 3 group. ^ο^ *P* > .05, ***P < .001. Ovoid nucleus (white arrowhead), indented nucleus (black arrowhead), lobulated (white thin arrow), and multilobed nucleus (black thin arrow). **A**, **B**, **C**, and **D**: ×1000.

**Figure 9:**
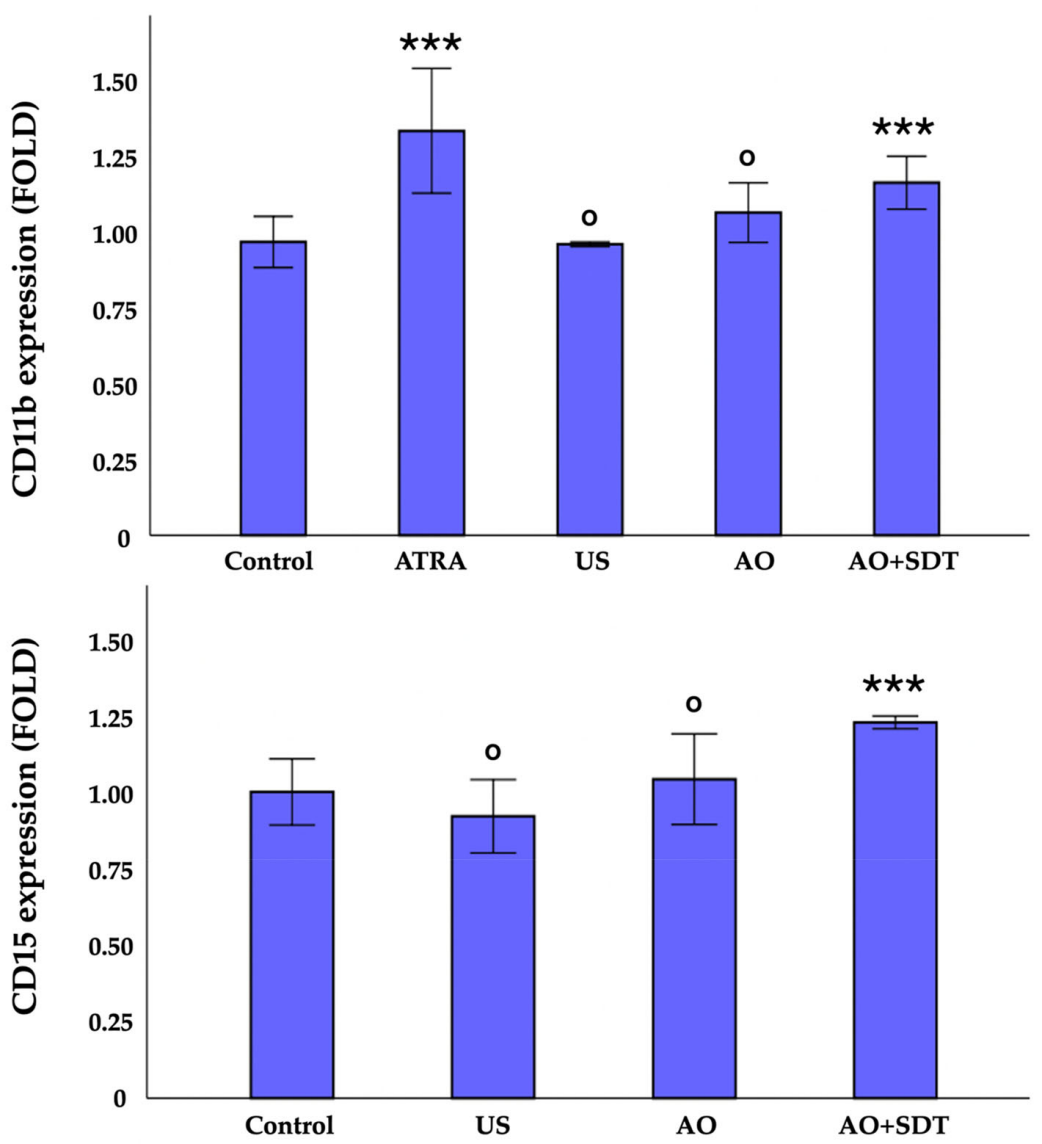
Cell surface antigen CD11b and CD15 expression analysis by flow cytometry. HL60 cells were plated at a density of 5 × 10^5^ cells/mL, and cultured for 72 hours. AO‐mediated SDT significantly increased the number of CD11b antigen expressing HL60 cells in IC_50_ value. However, only AO and US group had weak or virtually no such potency in increasing CD11b and CD15 antigen‐expression even at a concentration up to IC_50_ value. Control: untreated cells. Values represent the mean ± SD of data from 3 different experiments. *** indicates statistically significance compared to control group; ^ο^ indicates not statistically significance compared to control group. ^ο^ *P* > .05, ***P < .001.

In the examination of cell morphology performed on semithin sections stained with toluidine blue, the majority of cells in the control group exhibited ovoid nuclei with a normal appearance. The chromatin was uniformly distributed within the nucleus, and the nucleolus was prominently visible (Figure 8A). Statistically significant differences were identified in terms of nuclear differentiation indicators, such as ovoid, indented, lobulated, and morphology, in all groups (*P* < .001), except for the multilobed nuclear morphology in the US and AO groups (*P* = .63) (Figure 8, B and C) and the indented nuclear morphology in the control and US groups (*P* = .026) (Figure 8, A and B). These structures gradually increased in the control, US, AO, and AO‐mediated SDT groups, respectively, while the ovoid structure, which is the normal nuclear morphology, showed a gradual decrease. Consequently, a significant increase in cell differentiation was noted (Figure 8, D and E). ATRA is an agent that provides differentiation in the treatment of APL.^35^ Therefore, it was used as a positive control. Expressions of CD11b and CD15 antigens were used to examine myeloid differentiation of HL60 cells. As shown in Figure 9, AO‐mediated SDT increased the number of CD11b (1.3‐fold) and CD15 (1.2‐fold) antigen expressing cells within a IC_50_ concentration compared to control cells. These findings demonstrate that AO‐mediated post‐SDT can induce differentiation of HL60 cells along the granulocytic lineage. While CD11b and CD15 antigen expressions were statistically significant in the ATRA (CD15 antigen expression data of the ATRA group cannot be shown due to a technical error) and AO‐mediated SDT experimental group compared to the control group (*P* < .001), no significant change was observed in the expressions of differentiation markers in the AO‐only group and the US group compared to the control group (*P* > .05). As a result, the examination of morphology with toluidine blue and the CD11b, CD15 expression analysis were used as another evaluation of granulocytic differentiation after AO‐mediated SDT treatment of HL60 cells. The purpose of these experiments was to define the 4 nuclear morphologies: oval, indented, lobed, and multilobed during granulocytic differentiation, and to determine whether there was an increase in the expression of cell surface antigens. Differentiation was confirmed by observing the expected changes in nuclear morphology and the expected changes in the expression of cell surface antigens CD11b and CD15.

## Discussion

In vitro experiments play a crucial role in unraveling the mechanisms underlying cell death following SDT. Numerous studies suggest that the mechanisms of cell death by SDT closely resemble those in PDT. SDT‐induced apoptosis has been documented across various cell types using a range of sensitizers.^11^, ^18^, ^36^, ^37^, ^38^, ^39^, ^40^ Furthermore, the combination of sonosensitizers and ultrasound has been shown to trigger apoptosis in leukemia cells. Alterations such as damage to the cell membrane, mitochondrial dysfunction, chromatin condensation, and increased intracellular ROS production resulting from SDT have been identified as critical factors inhibiting leukemia cell growth and even inducing cell death.^41^, ^42^, ^43^ However, the mechanism of action of AO‐mediated SDT on HL60 cells remains unclear. This study investigated the impact of AO in combination with ultrasound stimulation on HL60 acute promyelocytic leukemia cells.

The assessment of HL60 cell viability after AO‐mediated SDT was conducted using the MTT assay. AO‐mediated SDT exhibited a significantly higher cytotoxic effect compared to both the US treatment alone and the AO treatment alone. These results indicate that AO may have the potential to serve as an effective sensitizer, and AO‐mediated SDT could represent a promising approach to combating leukemia.

The use of the electron microscopy technique is regarded as the “gold standard” for identifying apoptotic cells.^44^ During apoptosis, distinctive morphological changes occur, including nucleus shrinkage, nuclear marginalization, and chromatin condensation.^45^, ^46^ The nucleus fragments, the cytoplasm undergoes condensation, and the structure of other organelles changes.^47^ Apoptosis can be initiated through cell membrane receptors, depending on the inducing factor, or it can also occur due to an increase in ROS and calcium ions in the cytoplasm, leading to mitochondrial inactivation, DNA damage, oxidative stress, and disruptions in electrolyte transport.^46^ In contrast to the other groups, the presence of marked chromatin condensation, marginalization, lobulation, and apoptotic micronuclei in the nucleus, which are indicative of apoptosis, are prominently observed in the AO‐mediated SDT group. This underscores that AO‐mediated SDT robustly triggers apoptosis. Additionally, features such as the loss of mitochondrial cristae,^48^ mitochondrial vacuoles, and degenerative changes in many cells suggest the activation of the intrinsic apoptosis pathway. Furthermore, lipid droplets,^49^ loss of microvilli,^50^ and the presence of intracellular vacuoles,^51^ which are all characteristic indicators of apoptosis, were observed in numerous cells. In the US group, some cells displayed a loss of cristae, while in the AO group, apart from a few cells showing nuclear lobulation, a normal morphology similar to the control group was observed.

Annexin V and PI staining in flow cytometry is an important method for differentiating between viable, necrotic, early apoptosis, and late apoptosis in cells. In normal cells, phosphatidylserine, one of the membrane lipids, is typically located on the cytoplasmic side of the cell membrane. If the cell undergoes apoptosis, phosphatidylserine molecules, which are normally on the inner surface, are translocated to the outer surface of the cell membrane. This translocation occurs in the early stages of apoptotic cell death when cell membrane integrity remains intact. Annexin V, being a protein capable of binding to translocated phosphatidylserine on the outer cell surface, is a crucial tool for detecting early‐stage apoptosis. By introducing PI to cells stained with Annexin V, it becomes possible to identify necrotic and late apoptotic cells.^52^ This study demonstrated that 24.3% of the AO‐mediated SDT‐treated cells were in early apoptosis, 13.9% were in late apoptosis, and 7.9% were necrotic, indicating the presence of early‐stage apoptosis.

The subG1 results reveal that AO‐mediated‐SDT induces cell cycle arrest in the subG1 phase. These results suggest that the administration of AO‐mediated‐SDT holds crucial potential as a therapeutic approach for leukemia.

FSC values have been effectively employed in studies to detect cell shrinkage.^53^ This study established that AO‐mediated SDT resulted in a decrease in cell volume in HL60 cells, indicating an ongoing apoptotic process.

Mitochondria‐mediated apoptosis is triggered by a wide range of stimuli. The decrease in Δψm results from mitochondria depolarization, which is associated with apoptosis. Changes in mitochondrial membrane potential in HL60 cells after AO‐mediated SDT were monitored using flow cytometry. The results indicated that this treatment induced mitochondrial membrane depolarization, reduced cell viability, and ultimately led to apoptosis, resulting in significant mitochondrial damage following the treatment. There exists a crucial interplay between mitochondria and the production of ROS. ROS are highly reactive molecules primarily generated from the mitochondrial electron transport chain. When mitochondrial damage occurs, ROS production is activated within the mitochondria, potentially exacerbating the damage.^54^ Certain ROS by products are known to originate from sonosensitizers during the sonodynamic treatment process, contributing to cell death.^24^, ^42^, ^55^ In our study, ROS levels increased significantly following AO‐mediated SDT treatment in HL60 cells, whereas ROS formation was not observed in the control group. Consequently, excessive intracellular ROS production was induced by AO‐mediated SDT, implicating an oxidative stress mechanism in response to sonochemical effects in HL60 cells. The substantial production of ROS resulting from AO‐mediated SDT suggests the induction of cytoplasmic damage.

Numerous studies have demonstrated the induction of apoptosis following PDT in acute myeloid leukemia cells.^56^, ^57^, ^58^, ^59^, ^60^, ^61^, ^62^, ^63^ However, there are fewer studies elucidating the mechanism of action of sonosensitizer‐mediated SDT on HL60 cells. Su et al^64^ reported an increase in cytotoxicity of HL60 cells after protoporphyrin‐mediated SDT. According to the results of Annexin V‐PE/7‐AAD staining, significant induction of HL60 cell apoptosis was observed, and intracellular ROS production increased. Li et al^65^ reported that PpIX‐mediated ultrasound treatment in the human leukemia cell line U937 significantly reduced cell viability, causing substantial damage to cell morphology, DNA, and mitochondria, with intracellular ROS involvement.^66^ Another study showed that the growth of K562 cells was suppressed, intracellular ROS production increased, and mitochondria‐ and caspase‐dependent apoptosis was triggered after Ce6‐mediated SDT. Yumita et al^67^ reported the induction of apoptosis characterized by cell shrinkage, DNA fragmentation, and caspase 3 activation following fullerene‐mediated SDT in HL60 cells. Similar to these studies, our study revealed the induction of apoptosis following AO‐mediated SDT.

Another approach emerging in therapeutic strategies is differentiation therapy. This method involves the use of external agents, which can be either physical or chemical, to guide immature cells toward natural differentiation by modulating specific signaling pathways. Differentiation therapy is being seriously considered as a potential alternative in the treatment of certain conditions. The most successful example of differentiation therapy is the introduction of ATRA and arsenic trioxide (ATO) in the treatment of APL. Although disease‐free survival success has reached 80%–90%, relapse occurs in 5%–10% of patients due to resistance to ATRA or ATO.^33^ Therefore, there is a need to research new differentiation inducers with fewer side effects. SDT is a targeted therapy that includes a sonosensitizer, ultrasound, and oxygen, demonstrating immunomodulatory activity in both in vitro experiments and in vivo models.^58^, ^59^, ^60^, ^61^, ^62^, ^63^, ^64^, ^65^, ^66^, ^67^, ^68^, ^69^, ^70^, ^71^, ^72^ In vivo and in vitro studies have shown the effectiveness of SDT in the treatment of various diseases, including cancer, bacterial, parasitic infections, and others.^73^, ^74^, ^75^ In this study, the differentiation potential of AO‐mediated SDT in HL60 cells has been demonstrated. It is clearly evident that AO‐mediated SDT can induce differentiation in HL60 cells. Interestingly, our results have demonstrated that AO‐mediated SDT can suppress HL60 cell proliferation and induce differentiation. Additionally, AO‐mediated SDT has shown significant inhibitory effects on HL60 cell proliferation. AO‐mediated SDT has been demonstrated to induce HL60 cells toward the granulocytic pathway, as evidenced by the expression of CD11b and CD15 surface antigens, morphological features, and the presence of lobular morphology. In the literature, granulocytic differentiation resulting from AO‐mediated SDT can be compared with the results observed with ATRA, which is known as the most successful among differentiation inducers.^35^ Induction of differentiation of HL60 cells by AO‐mediated SDT in IC_50_ concentration supports the response similar to that of HL60 retinoic acid a differentiation inducer.

This research has some limitations. Currently, due to the variability of parameters such as power, frequency, intensity, and ultrasound pressure, a standard application procedure for SDT has not yet been established. The accumulation of data from many similar studies in this field will help solve these problems and accelerate the clinical translation of SDT. Another limitation of this study is that AO‐mediated SDT against HL60 cells is based on an in vitro design. The accuracy of our results should be verified with in vivo studies. Therefore, it is appropriate to evaluate the results obtained from the analyses in light of some limitations. However, our study also has some strengths. Besides contributing to the literature with the optimization of ultrasound application, AO‐mediated SDT performed on HL60 cells provides valuable information in both suppressing cell proliferation and inducing differentiation. Despite observed differences in reviewed articles, valuable trends can be identified to guide future studies in sonodynamic anticancer therapy.

## Conclusion

In conclusion, the observed alterations in cell morphology, including chromatin condensation in the nucleus, marginalization, lobulation, the presence of apoptotic micronuclei, and the loss of mitochondrial cristae, coupled with the presence of various apoptotic markers such as increased mitochondrial membrane depolarization, cell cycle arrest in the subG1 phase, reduced cell volume, early onset of apoptosis, and ROS release, collectively suggest a promising avenue for a cost‐effective, nontoxic, and noninvasive treatment for HL60 cells. According to all the results, it was observed that proliferation in HL60 cells was significantly inhibited after AO‐mediated SDT. Additionally, it was demonstrated that it could induce substantial differentiation. Our findings provide evidence that AO‐mediated SDT not only serves as an immunomodulator in cancer differentiation therapy but also holds potential as a chemotherapeutic agent in the treatment of APL.

## Compliance With Ethical Standards

This work was carried out without involving human participants or animals as objects of research.

## Supporting information


**Supplement S1** Supplementary Information.

## Data Availability

The data that support the findings of this study are available from the corresponding author upon reasonable request.
