# Supplementary material for: Monitoring Apoptosis and Myeloid Differentiation of Acridine Orange‐Mediated Sonodynamic Therapy‐Induced Human Promyelocytic Leukemia HL60 Cells
Source: J Ultrasound Med. 2024 Sep 10;44(1):15–34. doi: 10.1002/jum.16575 (PMC11632649; doi:10.1002/jum.16575)
Supplement: Supplementary file 1 — Supplement S1 Supplementary Information. [file JUM-44-15-s001.docx]

**ANOVA**

| **ANOVA** | | | | **Tukey HSD** | | | | | | | | | | | **Tamhane** |
| --- | --- | --- | --- | --- | --- | --- | --- | --- | --- | --- | --- | --- | --- | --- | --- |
|  |  |  |  | **SubG1** | **ΔΨm** | **CD15** | **Annexin/PI** | | | | **Toludine Blue staining** | | | | **Cell**  **volume** |
|  |  |  |  |  |  |  | Viable | Early  apoptotic | Late  apoptotic | Necrotic | Ovoid | Intented | Lobulalated | Multilobed |  |
|  | **Control** | | **US** | ,979 | 1,000 | ,204 | ,970 | ,985 | ,999 | 1,000 | **,000*** | ,026 | **,000*** | **,000*** | ,767 |
|  |  |  | **AO** | **,000*** | ,125 | ,668 | ,011 | ,288 | ,029 | ,601 | **,000*** | **,000*** | **,000*** | **,000*** | **,002*** |
|  |  |  | **AO+SDT** | **,000*** | **,000*** | **,001*** | **,000*** | **,001*** | **,005*** | ,037 | **,000*** | **,000*** | **,000*** | **,000*** | **,000*** |
|  | **US** | | **Control** | ,979 | 1,000 | ,204 | ,970 | ,985 | ,999 | 1,000 | **,000*** | ,026 | **,000*** | **,000*** | ,767 |
|  |  |  | **AO** | **,000*** | ,116 | ,041 | ,019 | ,435 | ,036 | ,653 | **,000*** | **,005*** | **,000*** | ,630 | ,046 |
|  |  |  | **AO+SDT** | **,000*** | **,000*** | **,000*** | **,000*** | **,002*** | **,006*** | ,042 | **,000*** | **,000*** | **,000*** | **,000*** | ,017 |
|  | **AO** | | **Control** | **,000*** | ,125 | ,668 | ,011 | ,288 | ,029 | ,601 | **,000*** | **,000*** | **,000*** | **,000*** | **,002*** |
|  |  |  | **US** | **,000*** | ,116 | ,041 | ,019 | ,435 | ,036 | ,653 | **,000*** | **,005*** | **,000*** | ,630 | ,046 |
|  |  |  | **AO+SDT** | **,000*** | **,000*** | **,004*** | **,002*** | ,014 | ,545 | ,217 | **,000*** | ,026 | **,001*** | **,000*** | **,001*** |
|  | **AO+SDT** | | **Control** | **,000*** | **,000*** | **,001*** | **,000*** | **,001*** | **,005*** | ,037 | **,000*** | **,000*** | **,000*** | **,000*** | **,000*** |
|  |  |  | **US** | **,000*** | **,000*** | **,000*** | **,000*** | **,002*** | **,006*** | ,042 | **,000*** | **,000*** | **,000*** | **,000*** | ,017 |
|  |  |  | **AO** | **,000*** | **,000*** | **,004*** | **,002*** | ,014 | ,545 | ,217 | **,000*** | ,026 | **,001*** | **,000*** | **,001*** |
|  | | *. The mean difference is significant at the 0.008 level. | | | | | | | |  |  |  |  |  |  |

| **ANOVA Tukey HSD**  **CD11b** | **Control** | **ATRA** | **US** | **AO** | **AO+SDT** |
| --- | --- | --- | --- | --- | --- |
| **Control** | **-** | **,000*** | 1,000 | ,155 | **,003*** |
| **ATRA** | **,000*** | - | **,000*** | **,000*** | ,008 |
| **US** | 1,000 | **,000*** | - | ,115 | **,002*** |
| **AO** | ,155 | **,000*** | ,115 | - | ,143 |
| **AO+SDT** | **,003*** | ,008 | **,002*** | ,143 | - |

*. The mean difference is significant at the 0.005 level.

**Independent sample t-test**

| **Independent sample test**  **Cell viabilty** | | **Control**  **&**  **US** | **AO Concentrations** | | | | | | |
| --- | --- | --- | --- | --- | --- | --- | --- | --- | --- |
|  |  |  | **0.0625**  **μM** | **0.125**  **μM** | **0.25**  **μM** | **0.5**  **μM** | **1**  **μM** | **2**  **μM** | **4**  **μM** |
| **AO 24** | **AO 48** | >,05 | ,248 | ,228 | ,198 | ,235 | ,155 | ,156 | ,318 |
|  | **AO 72** | >,05 | ,843 | ,904 | ,369 | ,226 | ,571 | ,536 | ,188 |
|  | **AO SDT 24** | >,05 | **,001**** | **,001**** | **,000***** | **,000***** | **,000***** | **,000***** | **,000***** |
|  | **AO SDT 48** | >,05 | **,000***** | **,000***** | **,000***** | **,000***** | **,000***** | **,000***** | **,000***** |
|  | **AO SDT 72** | >,05 | **,000***** | **,000***** | **,000***** | **,000***** | **,000***** | **,000***** | **,000***** |
| **AO 48** | **AO 72** | >,05 | ,166 | ,201 | ,620 | ,580 | ,500 | ,197 | ,311 |
|  | **AO SDT 24** | >,05 | **,000***** | **,000***** | **,000***** | **,000***** | **,000***** | **,000***** | **,000***** |
|  | **AO SDT 48** | >,05 | **,000***** | **,000***** | **,000***** | **,000***** | **,000***** | **,000***** | **,000***** |
|  | **AO SDT 72** | >,05 | **,000***** | **,000***** | **,000***** | **,000***** | **,000***** | **,000***** | **,000***** |
| **AO 72** | **AO SDT 24** | >,05 | **,001**** | **,001**** | **,000***** | **,000***** | **,000***** | **,000***** | **,000***** |
|  | **AO SDT 48** | >,05 | **,000***** | **,000***** | **,000***** | **,000***** | **,000***** | **,000***** | **,000***** |
|  | **AO SDT 72** | >,05 | **,000***** | **,000***** | **,000***** | **,000***** | **,000***** | **,000***** | **,000***** |
| **AO SDT 24** | **AO SDT 48** | >,05 | **,017^†^** | **,018^†^** | **,004^††^** | **,004^††^** | **,000^†††^** | **,000^†††^** | **,000^†††^** |
|  | **AO SDT 72** | >,05 | **,045^†^** | **,015^†^** | **,004^††^** | **,003^††^** | **,000^†††^** | **,000^†††^** | **,000^†††^** |
| **AO SDT 48** | **AO SDT 72** | >,05 | ,981 | ,377 | ,232 | ,329 | ,456 | ,564 | ,386 |

*. P < 0.05, **. P < 0.01, ***. P < 0.001

| **Independent Samples Test**  **AO+SDT compared to Control** | | Levene's Test for Equality of Variances | | t-test for Equality of Means | | | | | | | |
| --- | --- | --- | --- | --- | --- | --- | --- | --- | --- | --- | --- |
|  |  | F | Sig. | t | df | Sig.  (2-tailed) | Mean  Difference | Std. Error Difference | 95% Confidence Interval of the Difference | |  |
|  |  |  |  |  |  |  |  |  | Lower | Upper |  |
| **ROS** | Equal variances assumed | 2,481 | ,190 | -107,327 | 4 | ,000 | -30,56667 | ,28480 | -31,35740 | -29,77593 |  |
|  | Equal variances not assumed |  |  | -107,327 | 3,041 | ,000 | -30,56667 | ,28480 | -31,46619 | -29,66715 |  |
